# The Dental Profession

**Published:** 1873-01

**Authors:** 


*The Dental Profession.*—Dr. O. Willson, President of the "Illinois State Dental Society," in an address delivered at the recent meeting, remarks as follows:

"No profession, perhaps, in proportion to its numbers and intelligence, contributes more largely to health than does ours, because the arena of its *labors* possesses the *widest possible range* of sympathy with the rest of the body; nevertheless, this demand cannot be by reason of the great number of intelligent dentists, diffusing this knowledge among the people. Although all knowledge does not originate in colleges, (though in some instances it does, and remains there,) to our shame be it remembered that not more than one-fifth of our profession in active practice in this country are graduates of dental colleges, and there are no dental colleges elsewhere (that we know of.) The medical profession count, say 75,000 members, of whom nine-tenths possess diplomas from respectable medical schools. So long as this condition of things exists, we can hardly look for the cause of the demand in this direction. If it were because of the rapid increase of the tooth prizing population, then the ratio in numbers, of dentists and people, would keep more even pace—a conclusion not warranted by our own observation. If, then, the rapid growth of our specialty cannot be attributed solely to any one of the causes mentioned, all may, more or less, contribute; but especially the increased disturbed equipollence of the fluids of the mouth, and the want of normal formation, and resisting power, on the part of the teeth themselves.

"A consideration of the causes suggested by this latter condition leads into remote and exceedingly abstruse fields of research, perhaps more important than all—yet, when we contemplate the present condition of enlightened society, its tastes and hurry, the mind is at all times bewildered and the hands paralyzed.

"It is possible, however, that in the progress of dental science, remedy receives more attention than the cause which makes it necessary. Certainly the mechanical remedy has taken precedence, because it has seemed next to impossible to do better—upon which the question may well be raised, whether this is progress in its highest state?

"But while none feel like disparaging the wonderful attainments of the mechanical remedy, or retarding its further progress, all, perhaps, more and more feel the necessity of first removing the *cause* of disease; you have done much every way; you can and do arrest dental caries, and some already, having passed the abatis, are actually storming the citadel of disease, so far, as to say, that *no tooth should ever be extracted.*

"Artificial teeth vie with the natural for beauty and durability. You have given to the world the practical use of general anæsthetics, and if the dental profession had done nothing more, it would deserve, and will enjoy, the perpetual gratitude of humanity."

